# Challenges related to transitioning from hospital to temporary care at a skilled nursing facility: a descriptive study

**DOI:** 10.1007/s41999-024-01003-z

**Published:** 2024-06-15

**Authors:** Lene Vestergaard Ravn-Nielsen, Emma Bjørk, Marianne Nielsen, Stine Galsgaard, Anton Pottegård, Carina Lundby

**Affiliations:** 1 Hospital Pharmacy Funen, Odense University Hospital, Solfaldsvej 38, 5000 Odense C, Denmark; 2 Clinical Pharmacology, Pharmacy and Environmental Medicine, Department of Public Health, University of Southern Denmark, Odense, Denmark; 3 Department of Public Health, Research Unit of General Practice, University of Southern Denmark, Odense, Denmark

**Keywords:** Skilled nursing facility, Multimorbidity, Medication management, Continuity of care, Communication

## Abstract

**Purpose:**

With decreasing number of hospital beds, more citizens are discharged to temporary care at skilled nursing facilities, requiring increasingly complex care in a non-hospital setting. We mapped challenges related to the transition of citizens from hospital to temporary care at a skilled nursing facility in relation to medication management, responsibility of medical treatment, and communication.

**Methods:**

Descriptive study of citizens discharged from Odense University Hospital to temporary care from May 2022 to March 2023.

**Results:**

We included 209 citizens (53% women, median age 81 years). Most citizens (97%; *n* = 109/112) had their medication changed during hospital admission. Citizens used a median of eight medications, including risk medications (96%, *n* = 108). Medication-related challenges occurred for 37% (*n* = 77) of citizens and most often concerned missing alignment of medication records. Half of citizens (47%, *n* = 99) moved into temporary care with all medication needed for further dispensing. Nurses conducted in median three telephone calls (interquartile range [IQR 1–4]) and sent in median two correspondences (IQR 1–3) per citizen within the first 5 days. Nurses most often called the hospital physician (41% of telephone calls, *n* = 265/643) and sent correspondences to the general practitioner (55% of correspondences, *n* = 257/469). For 31% (*n* = 29/95) of citizens requiring action from nursing staff, this could have been avoided if the nurses had had access to the discharge letter.

**Conclusion:**

We identified several challenges related to the transition of patients from hospital to temporary care, most often related to medication. A third of actions related to medication management were considered avoidable with improved practices around communication.

## Key summary points

**Aim:**

To map challenges related to the transition of citizens from hospital to temporary care at a skilled nursing facility in relation to medication management, responsibility of medical treatment, and communication.

**Findings:**

Half of the citizens possessed all medication needed for further dispensing when they arrived at the skilled nursing facility. The nurses conducted in median three telephone calls and sent in median two correspondences per citizen.

**Message:**

A third of contacts related to medication management were avoidable with improved practices around communication.

## Introduction

Temporary care beds outside the hospital are critical subsystems in modern healthcare systems, providing short-term care and rehabilitation for patients, most often following hospital admissions [1]. These beds are increasingly important as the aging population continues to grow, placing greater demands on healthcare systems worldwide [2]. Temporary care beds can help alleviate the pressure on hospitals, freeing up resources for more urgent cases while providing patients with the necessary care and support to aid their recovery.

The high throughput of increasingly complex and frail patients into temporary care beds [3–5] constitutes a challenge for healthcare providers [4]. One of the primary issues faced is communication with other parts of the healthcare system, including hospitals, primary care providers, and long-term care facilities [6–9]. Effective communication is essential for ensuring continuity of care and avoiding adverse events such as medication errors, readmissions, and delays in care [7, 10, 11]. The challenges posed by communication breakdowns in the healthcare system can lead to negative outcomes for patients [11, 12] and increase the burden on healthcare providers [13, 14].

To improve care for the growing population of older people, it is essential to understand the challenges associated with the transfer of patients from the hospital to temporary care. In this study, we aimed to describe and systematically map these challenges related to the transition of patients from hospital to temporary care at a skilled nursing facility (SNF) in relation to medication management, responsibility of medical treatment, and communication.

## Methods

We conducted an observational study describing challenges related to the transition of patients being discharged from either a medical or surgical department at a large tertiary university hospital to short-term temporary care at a SNF.

### Setting

The study was conducted at Odense University Hospital and the SNF “Lysningen”, both located in the Region of Southern Denmark.

Odense University Hospital is a large tertiary university hospital with 965 beds, receiving patients mainly from Funen (approx. 500,000 citizens). The hospital discharges approx. 100,000 patients per year and has 42 medical and surgical departments. Of these, 19 discharges patients to “Lysningen” and were thus included in this study.

“Lysningen” is a SNF located in Odense Municipality. It is a large tertiary care center with 64 temporary care beds which provides care and treatment to patients who, following an admission, are not well enough to return to their home. Citizens are referred to such a stay by their municipality, based on a suggestion from the hospital in relation to discharge. Care is provided by nurses, healthcare assistants, physiotherapists, occupational therapists, and pharmaconomists. Politically, there are no demands of physicians in permanent positions at SNFs in Denmark [5]. Thus, medical treatment and changes to this is managed by the hospital physician during hospital admission, while it is the citizens’ general practitioner, i.e., the primary care setting, who is responsible for treatment and medication changes in the SNF. Care providers at “Lysningen” only get the discharge notice from the hospital nurses and not the discharge letter from the hospital physician. To prevent information gab, a phone call between a nurse from the SNF and a nurse from the hospital, coordinating citizens’ needs of care and treatment as well as practicalities before discharge, is made. Citizens are responsible themselves for bringing medication needed for further dispensing following the first 24 h after hospital discharge. The capture area for “Lysningen” is citizens from Odense Municipality (approx. 200,000 citizens).

### Study design

The study was an observational study conducted in a two-step design.

The first step provided a preliminary mapping and categorization of challenges related to the transition of patients from hospital to temporary care. A clinical pharmacist (LVRN) followed and observed nurses’ and pharmaconomists’ (comparable to a pharmacy technician, although with an education of 3 years) way of handling contacts to hospital physician, general practitioner, community pharmacy, and others involved in treatment and care, such as relatives, for 11 citizens, after which knowledge saturation was established (i.e., where no further information was obtained). The findings were discussed with a steering group supporting the study, comprising a general practitioner and a consultant from Odense Municipality, a consultant and a PhD student in emergency medicine from Odense University Hospital as well as a nurse from and the head of “Lysningen”. The project and steering group decided to further explore challenges related to medication management, responsibility of the medical treatment, and communication. For each of the three categories, factors associated with challenges related to the transition of patients from hospital to temporary care were identified among the project and steering group (as described in the data collection section).

The second step consisted of mapping the patients’ way through the system by a comprehensive systematic registration and categorization of the challenges described in the first step.

### Patients

Patients discharged from all discharging bed departments of Odense University Hospital were enrolled in the study. Patients were eligible if they were aged 18 years or above and could provide written informed consent. Initially, patients with dementia or other cognitive impairment were excluded because of inability to give consent. However, following approval from the Regional Council of Southern Denmark on October 17, 2022, these patients were included without providing written informed consent. Patients were censored if they left the SNF within 5 days of hospital discharge. This could be due to either being readmitted to the hospital, leaving by own choice, or dying. Based on the daily number of patients discharged from Odense University Hospital to “Lysningen” as well as the time limit of the study, we aimed for inclusion of 200 patients as this was considered feasible and sufficient to show any important tendencies.

### Data collection

Data collection took place from May 10, 2022 to March 10, 2023 and included consecutively discharged patients, hereafter referred to as citizens. Data were collected by trained pharmaconomists (MN and SG). For consistency, both data collectors were trained by the pharmacist carrying out the initial phase of the project. This entailed that the pharmaconomists followed the pharmacist during mapping of challenges for four citizens. Following this, the pharmaconomists had audit training sessions with the pharmacist. Data were collected every day between 8 am and 3 pm (weekends excluded) and were registered for the first 5 days of each citizen’s stay at the SNF. Data were collected from review of records in the interdisciplinary system NEXUS used in Odense Municipality as well as from consultation with the nurses and pharmaconomists working in the SNF. The following information was collected for each citizen: demographics, medication status, discharging department, residential information, medication administration status, level of function, changes of medication, number of telephone calls, electronically sent correspondences and to whom these were addressed, and an assessment of whether the required contacts could have been avoided if the nurses had had the discharge letter from the hospital physician and not only the usual discharge notice from the nurses. The medication list (excluding vitamins and nutritional supplements) was evaluated by number of changes during hospitalization and analyzed based on information from the Shared Medication Record and the electronic patient record. Risk medications were categorized inspired by Pirmohamed et al. [15] and modified to present medications available in Denmark. All data were processed in the online-based Research Electronic Data Capture (REDCap) system [16].

### Statistics

Descriptive statistics were used to analyze patient characteristics and the quantitative outcomes. All analyses were performed using Stata 17 (StataCorp, College Station, TX, USA).

### Ethics approval

Patient capable of providing written informed consent was included on this basis. Patients with dementia and other cognitive impairment were included without written informed consent after approval from the Regional Council of Southern Denmark (record no. 21/67763) on October 17, 2022. These citizens’ relatives were informed of the inclusion and had the opportunity to object. Before this date, these citizens were excluded. The study was registered in the Region of Southern Denmark’s repository (record no. 22/48161). The Regional Committee on Health Research Ethics waived registration (record no. 21/35007) due to the study design.

## Results

**Fig. 1:**
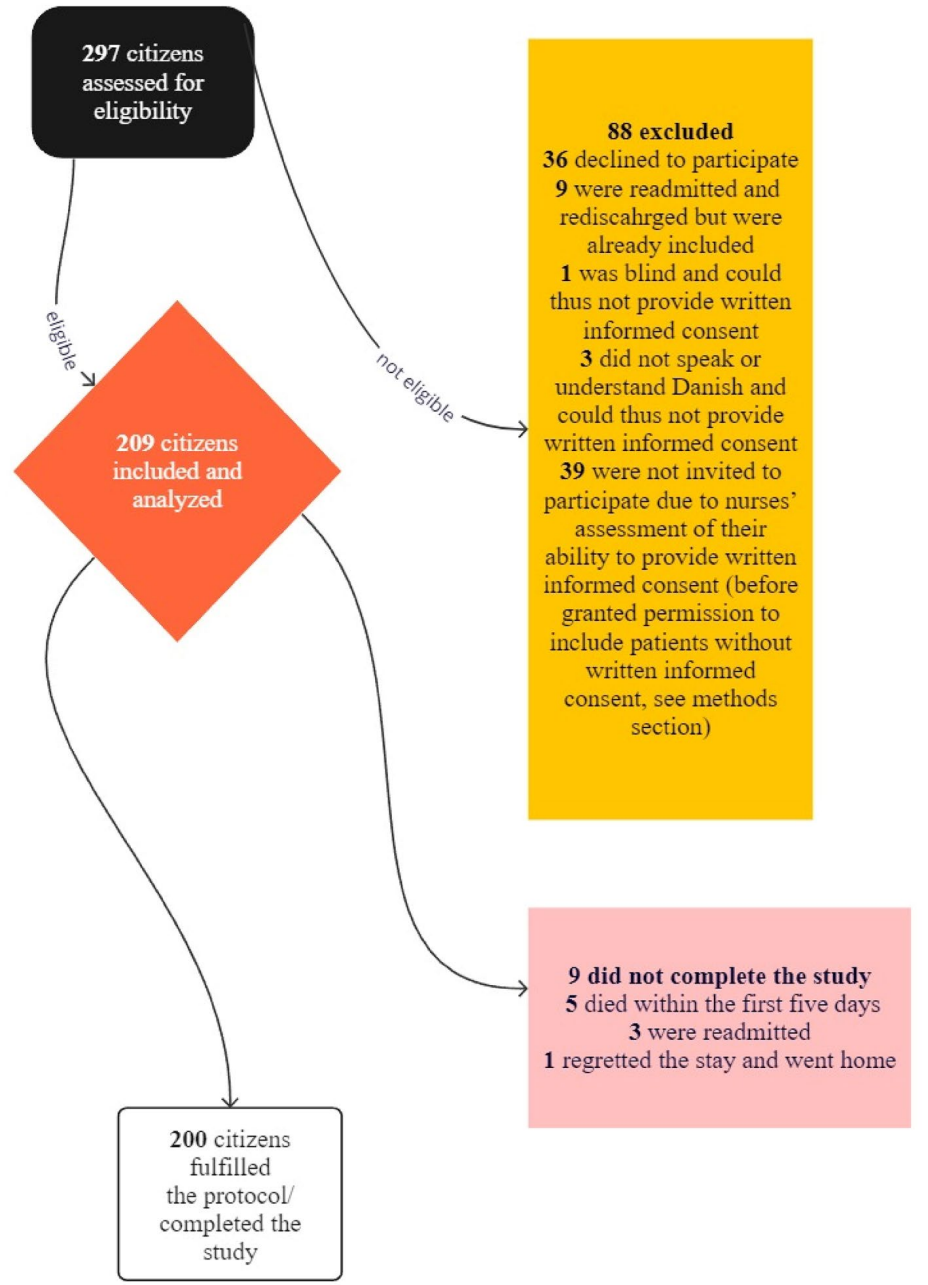
Flowchart of the 297 citizens invited to participate in the observational and descriptive study conducted with patients admitted to departments at Odense University Hospital and discharged to skilled nursing facility

A total of 297 citizens were invited to participate in the study, of which 209 were included (Fig. 1).

Following inclusion, nine citizens were censored during follow-up, of which five died during the first five days of their stay at the SNF, three were readmitted to the hospital, and one regretted their stay and went home.

Half of all included citizens (53%; *n* = 111) were women and the median age was 81 years (interquartile range [IQR 74–86]) (Table 1).

**Table 1:** Patient characteristics of citizens receiving temporary care at the skilled nursing facility “Lysningen”

*n* = 209	*n* (%)
Women	111 (53)
Age	
Median (IQR)	81 (74–86)
< 50 years	2 (1)
50–64 years	17 (8)
65–79 years	74 (35)
80 + years	116 (56)
Citizens with dementia or other cognitive impairment	39 (19)
Citizens with cardiopulmonary resuscitation assessment^a^	126 (60)
Citizens with terminal illness^a^	18 (9)
LOS in hospital, median (IQR)	9 (6–14)
Citizens discharged from medical wards	152 (73)
Citizens discharged from surgical wards	57 (27)
Level of function^b^	
No help	52 (25)
Mild level of help, e.g. cleaning, medication	40 (19)
Moderate to severe level of help, e.g. lifting, eating, clothing, etc.	117 (56)
Residential status^b^	
Care home/assisted living residence/skilled nursing facility	17 (8)
Own home	186 (89)
Other, e.g., homeless	6 (3)
Medication administration status^b^	
Self-administration	80 (38)
Dose drug dispensing	3 (1)
Medication management by nurse	112 (54)
Medication management by relative	14 (7)

*IQR* interquartile range, *LOS* length of stay

^a^Before hospital discharge

^b^Before hospital admission

One in five citizens (19%; *n* = 39) had dementia or otherwise impaired cognition. Citizens were mainly discharged from either the Geriatric Department (22%; *n* = 45), Neurological Department (17%; *n *= 36), or Orthopedic Department (13%; *n* = 28).

Around half of citizens were transferred to the SNF on weekdays before 3 pm (54%, *n* = 112), with the remaining being transferred after 3 pm (46%, *n* = 97). Monday was the most frequent weekday for transfer (24%, *n* = 50). One in ten citizens (11%, *n* = 24 of 209) were transferred on either Friday after 3 pm or during the weekend.

### Medication management

The majority (97%; *n* = 109/112) of citizens had their medication changed during hospital admission before arrival at the SNF. Most citizens (86%; *n *= 96) had one or more (median 2.9 [IQR 1–4]) new medications started, 23% (*n* = 26) had one or more (median 0.3 [IQR 0–0]) medications stopped, and 97% (*n* = 109) had changes in strength or dose to one or more (median 4.9 [IQR 3–7]) medications (data not shown).

Citizens were discharged from the hospital and arrived to the SNF with a median of eight medications (IQR 5–11) (Table 2).

**Table 2:** Number of medications at hospital admission and when arriving at the skilled nursing facility as well as risk medications categorized inspired by Pirmohamed et al. [15] and modified to present medications available in Denmark

*n* = 112^a^	
Medications at hospital admission, median (IQR)	7.8 (5–11)^a^
Medications at arrival to skilled nursing facility, median (IQR)	8.2 (5–11)^b^
Risk medication (ATC)	Citizens with medication at admission to skilled nursing facility, *n* (%)
Anticoagulants (B01AA.X, B01AB.X, B01AE.X, B01AF.X)	51 (46)
Antiplatelets (B01AC.X)	42 (38)
Angiotensin-converting enzyme (ACE)-inhibitors/ angiotensin II (AT-II) receptor antagonists (C09.X)	45 (40)
Beta blockers (C07.X)	36 (32)
Digoxin (C01AA05)	16 (14)
Diuretics (C03.X, C04B.X, C09D.X)	54 (48)
Antidepressants (N06A.X)	27 (24)
Opioids (N02A.X, R05DA04)	53 (47)
Nonsteroidal anti-inflammatory drugs (NSAIDs) (M01A.X)	*n* < 5
Steroids (H02.X)	11 (10)

*IQR* interquartile range, *ATC* Anatomical Therapeutic Chemical Classification System

^a^Because of implementation of a new electronic journal system, data were only available for 112 citizens at both admission and at discharge from hospital to the skilled nursing facility, therefore only those 112 citizens are used for comparison in Table 2

^b^209 citizens had data on number of medications at discharge from hospital to the skilled nursing facility

The majority of citizens (96%, *n* = 108) used at least one risk medication at time of arrival. The most common risk medications were diuretics (48%; *n* = 54), opioids (47%; *n* = 53), and anticoagulants (46%; *n* = 51). A sub-analysis for the group of citizens with impaired cognition showed no difference in relation to medication use (data not shown).

For almost all citizens (99%; *n* = 207), the medication provided by the hospital to the SNF was used during the first day. For 37% (*n* = 77) of citizens, there were problems related to the medication provided, most often due to missing update of the Shared Medication Record, change in strength of medication, missing or unidentified medication, or other discrepancies. Further, during the first day only half of citizens (47%; *n* = 99) either brought or were brought all medication needed for further dispensing for the first week and further during their temporary care stay.

### Responsibility of medical treatment

**Fig. 2:**
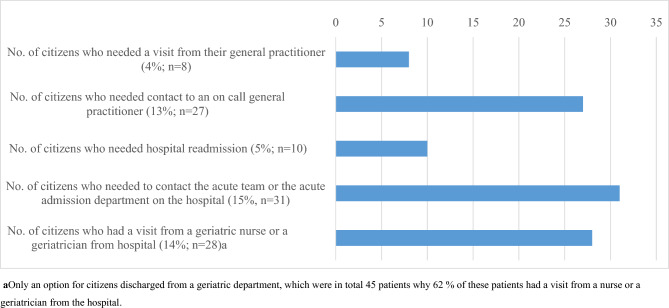
Number of citizens that need to contact or received a visit from a health-care provider at the skilled nursing facility, within the first 5 days. Type of healthcare provider is specified to general practitioner, on-call general practitioner, number of hospital readmissions, and acute team from hospital or geriatric nurse or a geriatrician from hospital. Some citizens received multiple visits or contacts per day but is only represented once in this figure

The type of healthcare-related contacts or visits are presented in Fig. 2.

During the first 5 days, the general practitioner visited 4% (*n* = 8) of citizens, the on-call general practitioner was contacted for 13% of citizens (*n* = 27), while the municipal acute team or acute admission department was contacted for 15% of citizens (*n* = 31). Fourteen percent (*n* = 28) had a follow-up visit from a geriatrician from the hospital and 5% (*n* = 10) of citizens were readmitted.

The citizens with impaired cognition had a similarly prevalence of contacts or visits within the first 5 days at the SNF (data not shown).

### Communication

The nurses conducted in median three (IQR 1–4) telephone calls and sent in median two electronically correspondences (IQR 1–3) per citizen about medication within the first 5 days (Table 3).

**Table 3:** Number of contacts by the nurses from the skilled nursing facility, specified within the first 5 days, topics of contacts and recipients of contacts. Several citizens needed more than one contact per day during the first 5 days

*n* = 209^a^	Telephone calls, *n* (%)	Electronic correspondences, *n* (%)	Combined contacts^b^, *n* (%)
Median number of contacts per patient within the first 5 days, (IQR)	3 (1–4)	2 (1–3)	5 (4–6)
Total number of contacts, *n*	643	469	1112
Topic of contact			
Medication, *n* (%)	364 (57)	297 (63)	661 (59)
Other^c^, *n* (%)	279 (43)	172 (37)	451 (41)
Recipient of contact			
Hospital staff, *n* (%)	265 (41)	40 (8.5)	305 (27)
General practitioner, *n* (%)	101 (16)	257 (55)	358 (32)
Private specialist, *n* (%)	2 (0.3)	2 (0.4)	4 (0.4)
On-call practitioner, *n* (%)	10 (1.6)	0 (0.0)	10 (0.9)
Community pharmacy, n (%)	68 (11)	162 (35)	230 (21)
Other^d^, *n* (%)	197 (31)	6 (1.3)	203 (18)

*IQR* interquartile range

^a^Only 200 of 209 citizens fulfilled all five days at the the skilled nursing facility

^b^Combined contacts are a combination of telephone calls and electronic sent correspondences

^c^Other includes blood samples, blood glucose levels, rehabilitation planning, ordering helping aids, scheduling appointment, oxygen treatment, ordering probe nutrition, make appointments with relatives in relation to assessment of assistive devices, telephone calls to a general practitioner according to concerns about diarrhea, rash and blood pressure

^d^Other include e.g. relatives, medical company about nutrition, etc.

Most telephone calls and correspondences were made on the second day (data not shown). It was primarily the hospital physician who was called and the general practitioner to whom the correspondences were addressed (Table 3). The contacts primarily concerned medication (Table 3). Coordinating telephone calls between the hospital nurse and the nurse from the SNF before hospital discharge were made for all 209 citizens.

We assessed 83% (*n* = 169 of 204) cases with discrepancy between the discharge notice from the nurses and the discharge letter, most often related to medication. In half (53%; *n *= 109 of 204) of cases with discrepancy, no action defined as phone call or correspondence was needed from the SNF to provide care and treatment. However, for 31% (*n *= 29 of the 95) of citizen records that required action from the SNF, it was estimated that the action could have been avoided if the nurses from the SNF had had the discharge letter.

A sub-analysis for the group of citizens with impaired cognition showed no difference in relation to communication (data not shown).

## Discussion

We identified cross-sectorial challenges related to the transition of patients from hospital to temporary care at a SNF. Citizens moving into temporary care used multiple medications, most often including risk medications. Cross-sectorial challenges most often concerned lack of needed medication for dispensing at the SNF, meaning that the nurses at the SNF had to repeatedly contact primary care and hospital physicians. A third of all actions related to medication management could have been avoided with improved communication, with nurses at the SNF having access to the necessary information acquired from the journals, particularly the discharge letter.

Citizens arrived at the SNF with a median of eight medications which is similar to a study from the US [17]. Further, most citizens were treated with risk medications [15] as seen in another Danish study with similar older citizens [18]. Risk medications make these citizens a vulnerable group with high risk of hospital admissions [19].

In Denmark, we use the Shared Medication Record which is a personal profile for the individual citizen that provides full access to current medication, updated by the physician who last treated the citizens [20]. Unfortunately, this record is not always updated. The findings from our study are aligned with discrepancies reported in other studies [21, 22]. The resulting recurrent contacts to hospital or general practitioner are very time-consuming for the nurses at the SNFs. Furthermore, uncertainty about whether it is the right medication or not can compromise patient safety and lead to considerable frustration for the healthcare providers [23]. Alignment of medication currently often requires involvement of relatives as medication being obtained is based on the resources and availability of the relatives rather than the IT system and organizationally workflows**.**

The nurses from the SNF called the hospital physician mostly on the second day of the citizens stay. This is likely explained by the fact that 40% of patients arrived after 4 pm, i.e., where the staffing is lower, and the general practitioners are unavailable. The timing of discharge and transition to the SNFs was also highlighted as a problematic theme in recent a qualitative study [24]. Further, the telephone calls on the second day supports the national Danish decision of the responsibility of the citizens’ treatment being upheld by the hospital for 72 h after discharge. At the time of the study, this was not implemented in the region where the study took place. There were no physicians at this SNF, which is why the general practitioners and hospital physicians were contacted. Further, implementation of designated general practitioners at care homes in Denmark has been shown to reduce the number of acute admissions, short-term admissions, and readmissions [25], while it also contributes to more clear, precise, and timely communication between care homes and the general practitioner [26] This indicates that it could be worth trying to have a designated general practitioner at the SNFs in Denmark. Finally, geriatric follow-up has been shown to reduce unplanned hospital readmissions in a similar study population [27]. In our study, six of ten citizens had a follow-up visit from a geriatrician, potentially preventing citizens from being readmitted to the hospital.

Four out of five citizens had one or more discrepancies between the discharge notice from the nurses at the hospital and the discharge letter. These discrepancies covered missing information about medication, changes of dose, time for treatment, control of blood samples, etc. As estimated, a significant proportion of all telephone calls and correspondences from the SNF to general practitioners and hospitals related to such missing information. This indicates that shared information, where the nurses at the SNF could access all the information from the electronic patient journal from the hospital, would be valuable. Specifically, the nurses should systematically receive the discharge letter with medical changes described. Several solutions to improve medication changes at transitions have been demonstrated [18, 28, 29]. Potential strategies for improving communication and patient care may as well be addressed in a future study.

Among the principal strengths of our study is its fairly large size and the possibility to access data across sectors and different systems, thus enabling us to achieve valid data. Second, this study includes citizens with dementia and other cognitive impairments, although only for a part of the study period. Several limitations to our study should also be acknowledged. First, citizens were only included once, even if the citizens had several hospital admissions and subsequently stays at the SNF. When several subsequent transfers occur across healthcare sectors it can result in more complications related to continuation of changed medications, lack of information, etc., which might result in an even higher need for the nurses at the SNFs to contact hospital and general practitioners. Second, number of telephone calls and electronic correspondences were based on recollection by SNF staff or registrations in journals. However, the healthcare staff is very busy, and it is possible that not all contacts were reported, hereby leading to an underestimation of the number of contacts. Third, the heterogeneous study population includes not only older citizens but also a few younger and homeless citizens with, e.g., psychical illness and abuse. This heterogeneity makes it difficult to generalize the results. Finally, we collected data from one municipality with one SNF, which might also limit generalizability.

Generally, the outcome of a temporary stay is questioned in another Danish study which found varying effect [30], and the organization is of great political interest in these years, questioning how the citizens get the best treatment with most value for money.

## Conclusions

Most citizens experienced polypharmacy, most often including risk medications, when transitioning into temporary care at the skilled nursing facility. We identified challenges related to particularly lack of needed medication and found that the nurses from the SNF had to contact hospital physicians and primary care repeatedly, particularly on the second day. A third of the actions related to medication management were considered avoidable with improved practices around communication.

## Data Availability

Data can be shared upon contact to the corresponding author and upon achieving the necessary approvals.
